# Long-term psychological outcome after discharge from intensive care

**DOI:** 10.5935/0103-507X.20180008

**Published:** 2018

**Authors:** Sara Pereira, Sara Cavaco, Joana Fernandes, Inês Moreira, Eduarda Almeida, Filipa Seabra-Pereira, Heloísa Castro, Maria de Jesus Malheiro, Ana Filipa Cardoso, Irene Aragão, Teresa Cardoso

**Affiliations:** 1 Intensive Care Unit, Hospital Santo António, Centro Hospitalar do Porto - Porto, Portugal.; 2 Neuropsychology Unit, Department of Neurology, Centro Hospitalar do Porto - Porto, Portugal.

**Keywords:** Cognitive dysfunction, Patient discharge, Quality of life, Anxiety, Depression, Stress disorders, post-traumatic, Intensive care units

## Abstract

**Objective:**

To investigate the longterm psychological outcome in survivors of critical illness after intensive care unit discharge.

**Methods:**

A prospective cohort of survivors admitted to a mixed intensive care unit between January and September 2010 was evaluated six months and five years after hospital discharge. The Dementia Rating Scale-2, the Hospital Anxiety and Depression Scale, the Posttraumatic stress syndrome 14-questions inventory, the Euro Quality of Life 5 Dimensions (EQ-5-D), and the Visual Analogue Scale (EQ VAS) were assessed at both follow-up periods.

**Results:**

Of 267 patients, 25 patients were evaluated at 6 months after discharge (62 ± 16 years); 12 (48%) presented cognitive impairment, 6 (24%) anxiety, 4 (16%) depression, and 4 (16%) post-traumatic stress disorder. Among those re-evaluated five years after discharge (n = 17; 65 ± 15 years), the frequency of cognitive impairment dropped from 8 (47%) to 3 (18%) (p = 0.063), due to improvement in these patients over time, and other patients did not acquire any dysfunction after discharge. At five years after discharge, only two patients (12%) reported anxiety, and none had depression or post-traumatic stress disorder. No differences were found between the six-month and five-year follow-ups regarding EQ-5-D and EQ VAS.

**Conclusion:**

Survivors do not show a progressive decline in cognitive function or quality of life within five years after intensive care unit discharge. Psychopathological symptoms tend to decrease with time.

## INTRODUCTION

Survivors of critical illness often have an incapacitating form of cognitive, psychological, and functional impairment, but the potential reversibility of these clinical conditions five years after intensive care unit (ICU) discharge remains inadequately characterized and understood.^(1)^ Cognitive dysfunction in this population is characterized by new deficits (or exacerbation of preexisting mild deficits) in global cognition, memory, attention/concentration, and executive functions. The etiology of cognitive impairment is dynamic and multifactorial, resulting from premorbid conditions and newly acquired brain injury due to insults associated with critical illness, such as hypoxia, glycemic dysregulation, hypotension, delirium, sedation and analgesic use.^(2,3)^ Profound and persistent deficits negatively impact the patients' functional and psychological status and health-related quality of life. Current evidence suggests that survivors may have persistent psychological morbidity, namely, anxiety and depression, compared with the general population.^(4)^

Previous data analyzing cognitive impairment were mainly based on follow-up periods between six months to two years post-ICU discharge^(1,5,6)^ or on a specific subset of patients.^(4,6-9)^ The main goal of our study was to prospectively evaluate the long-term cognitive function, mood, and quality of life of survivors of critical illness, at six months and five years after ICU discharge. The secondary goal was to identify predictive factors for cognitive dysfunction.

## METHODS

The prospective cohort consisted of survivors discharged from a 12-bed mixed ICU, in a 600-bed, tertiary care, university-affiliated hospital (*Hospital de Santo António, Centro Hospitalar do Porto*, Porto, Portugal) between January and September 2010. All patients were contacted for evaluation in the outpatient clinic. The exclusion criteria for the assessment were as follows: a previous neurological disorder, a recent coronary artery bypass revascularization or cardiac arrest, dependence for routine daily activities prior to ICU or at the time of the assessment, less than 3 years of education, and residence outside the hospital district (Porto).

Participants were evaluated at six months and at five years after hospital discharge, in the outpatient clinic, by an intensive care physician, a nurse and a psychologist. Cognitive impairment was studied using the Dementia Rating Scale-2 (DRS-2), a comprehensive and validated neuropsychological battery for the evaluation of global cognition, including attention, initiation/perseveration, visuospatial construction, conceptualization, and memory. The 10th percentile of age and education normative data for the Portuguese population was the cut-off used to detect cognitive impairment in the DRS-2.^(10)^

Psychopathology was evaluated with two self-report screening questionnaires, namely, the Hospital Anxiety and Depression Scale (HADS) and the Post-Traumatic Stress Scale-14 (PTSS-14).^(11,12)^ The cut-off for anxiety and depression was HADS ≥11 for each subscale, and the cut-off for post-traumatic stress disorder (PTSD) was PTSS-14 ≥ 45.

Quality of life was assessed with the European Quality of Life 5 Dimensions (EQ-5-D) and the EQ visual analogue scale (EQ VAS).^(13)^

The following independent variables were explored as potential risk factors for cognitive impairment: sex, age, years of education, severity of acute illness, glycemic dysregulation, hypoxia, use of sedative or analgesic medications during hospitalization, and ICU length of stay (LOS).

The study was approved by the Clinical Investigation Coordinating Department of the hospital (which includes the Ethics Committee of *Hospital de Santo António, Centro Hospitalar do Porto*) under nº 338-13. The study complied with the Declaration of Helsinki, and informed consent was obtained from all patients included.

### Statistical analysis

Continuous variables are described as the means and standard deviations (SDs) if they showed a normal distribution or as medians and inter-quartile ranges (IQRs) if they showed a skewed distribution. Categorical variables were described with absolute frequencies and percentages. The Wilcoxon test and McNemar test were used for paired comparisons. A simple logistic regression was applied to explore the predictors of cognitive impairment. The significance level was defined as p < 0.05. Data were analyzed using Statistical Package for Social Science (SPSS), version 23.

## RESULTS

**Figure 1:**
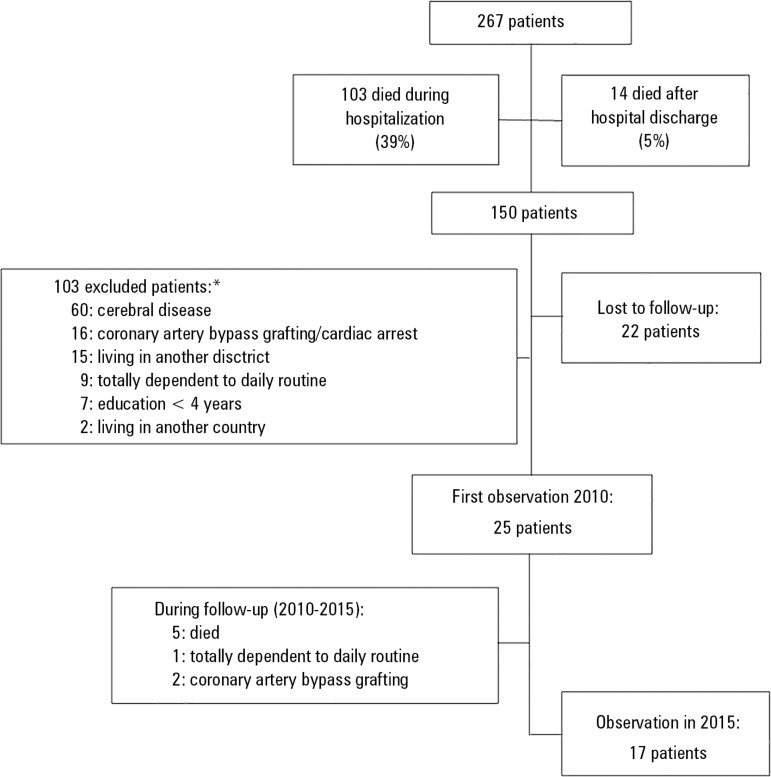
Flowchart of included patients. * Some patients met more than one of the exclusion criteria.

Of the 267 patients admitted to the ICU during the study period, 150 survived (56%), and 25 (17% of the survivors) met the inclusion criteria and were evaluated at the outpatient clinic at six months after hospital discharge (Figure 1). The general characteristics of the included patients are shown in table 1.

**Table 1:** Demographic and clinical characteristics of the included patients

Characteristics	Total (n = 25)	Extended follow-up (n = 17)
Sex (males)	14 (56)	7 (41)
Age (years)	62 ± 16	66 ± 15
Years of education	4 (4 - 8)	4 (4 - 12)
Type of admission		
Medical	14 (56)	10 (59)
Trauma	4 (16)	1 (6)
Non-elective surgery	7 (28)	6 (35)
SAPS II score	44 ± 15	45 ± 16
Respiratory SOFA	3 (2 - 4)	2 (2 - 4)
Cardiac SOFA	2 (0 - 2)	2 (0 - 3)
Glycemic dysregulation		
Glycemia < 40mg/dL	0 (0)	0 (0)
Glycemia > 180mg/dL	16 (64)	11 (65)
Severe hypoxia during ICU stay	8 (32)	6 (35)
Use of sedatives, analgesics or paralytics		
Propofol	9 (36)	5 (29)
Midazolam	19 (76)	13 (77)
Opioids	22 (88)	14 (82)
Paralytics	3 (12)	1 (6)
Days on sedatives and analgesics		
Sedatives	3 (2 - 11)	3 (2 - 11)
Analgesics	4 (1 - 11)	2 (1 - 11)
ICU length of stay	11 (4 - 22)	12 (5 - 27)

SAPS II - Simplified Acute Physiology Score II; SOFA - Sequential Organ Failure Assessment; ICU - intensive care unit. The results are expressed as the mean ± standard deviation, median (interquartile range) or n (%).

Of the initial cohort, during the five-year follow-up, five patients died, one became totally dependent for daily activities and two underwent heart surgery, leaving 17 patients to be re-evaluated (68% of the initial cohort). The demographic (i.e., sex, age, and education) and clinical (i.e., SAPS II score, respiratory SOFA, cardiac SOFA, glycemic dysregulation, severe hypoxia during ICU stay, use of sedatives, analgesics or paralytics, number of days on sedatives and analgesics and ICU length of stay) characteristics at the initial evaluation of those patients lost to follow-up were similar to those with two evaluations (p > 0.05), except for the presence of fewer men (41% *versus* 88%, p = 0.042) in the subgroup with the five-year evaluation. The frequencies of cognitive impairment, anxiety, and depression at six months were also not significantly different (p > 0.05).

Cognitive impairment was identified in 12/25 (48%) patients in the first evaluation (at the six-month follow-up from discharge). Among those re-evaluated five years after ICU discharge, the frequency of cognitive impairment dropped from 8/17 (47%) at six months to 3/17 (18%) at five years (p = 0.063). Of the first 12 patients with cognitive impairment at six months, four died, five recovered, and three maintained cognitive impairment at the five-year follow-up, translating into a 63% recovery rate (5/8 patients). The analysis of the five DRS-2 cognitive domains did not reveal significant changes between the two evaluations, except for a decline in the construction subscale (28% *versus* 59%; p = 0.031) (Table 2).

**Table 2:** Cognitive, psychological and physical function and quality of life evaluation 6 months and 5 years after intensive care unit discharge

Psychological measures	Total (n = 25)	Extended follow-up (n = 17)		p value
	6 months	6 months	5 years	
DRS-2 (< 10^th^ percentile)				
Total	12 (48)	8 (47)	3 (18)	0.063
Attention	13 (52)	9 (53)	6 (35)	0.508
Initiation/perseveration	6 (24)	2 (12)	0 (0)	NA
Construction	7 (28)	4 (24)	10 (59)	0.031*
Conceptualization	1 (4)	0 (0)	1 (6)	1.000
Memory	6 (24)	3 (18)	3 (18)	1.000
HADS				
Anxiety ≥ 11	6 (24)	3 (18)	2 (12)	1.000
Depression ≥ 11	4 (16)	0 (0)	0 (0)	NA
PTSS-14 (score ≥ 45)	4 (16)	2 (12)	0 (0)	NA
EQ-5-D (problems in)				
Self-care	7 (28)	3 (18)	4 (24)	1.000
Usual activities	12 (48)	7 (41)	6 (35)	1.000
Pain/discomfort	15 (60)	10 (59)	7 (41)	0.375
Mobility	15 (60)	8 (47)	8 (47)	1.000
Anxiety/depression	12 (48)	7 (41)	5 (29)	0.625
Visual analogue scale	60 (50 - 80)	60 (50 - 90)	75 (50 - 80)	0.599

DRS-2 - Dementia Rating Scale-2; HADS - Hospital Anxiety and Depression Scale; PTSS-14 - Post-traumatic stress syndrome 14-questions inventory; EQ-5-D - Euro Quality of Life 5 Dimensions; NA - not applicable.

* p < 0.05. The results are expressed as n (%) or median (interquartile range).

Regarding the predictors of cognitive impairment at the six-month follow-up from discharge, patients with episodes of hypoxia during ICU stay had less cognitive impairment than those without hypoxic events (Table 3). Patients with documented episodes of hypoxia during their ICU stay were younger (OR = 0.90, 95%CI: 0.82 - 0.98, p = 0.011) and tended to have less severe disease on admission, as measured by SAPS II (OR = 0.92, 95%CI: 0.84 - 1.00, p = 0.057). Older patients tended to have more cognitive impairment than younger patients. No significant association was found between cognitive impairment and the remaining demographic and clinical variables.

**Table 3:** Risk factors associated with cognitive impairment measured by a Dementia Rating Scale-2 < 10^th^ percentile

Variables	OR (95%CI)	p value
Sex (males)	2.33 (0.46 - 11.81)	0.306
Age (years)	1.06 (1.00 - 1.13)	0.054
Years of education	0.98 (0.81 - 1.18)	0.789
SAPS II score	1.01 (0.96 - 1.07)	0.744
Respiratory SOFA	1.13 (0.61 - 2.11)	0.696
Cardiac SOFA	1.36 (0.69 - 2.66)	0.373
Glycemia > 180mg/dL	2.57 (0.47 - 14.10)	0.277
Severe hypoxia during ICU stay	0.08 (0.01 - 0.79)	0.031
Use of sedatives, analgesics or paralytics		
Propofol	1.61 (0.31 - 8.32)	0.572
Midazolam	0.36 (0.05 - 2.50)	0.303
Opioids	NA	NA
Paralytics	0.50 (0.04 - 6.35)	0.59
Days on sedatives and analgesics		
Sedatives	1.011 (0.89 - 1.14)	0.867
Opioids	1.032 (0.91 - 1.17)	0.615
ICU length of stay	1.012 (0.98 - 1.05)	0.50

CI - confidence interval; SAPS II - Simplified Acute Physiology Score II; SOFA - Sequential Organ Failure Assessment; ICU - intensive care unit.

The HADS identified anxiety in 6/25 (24%) and depression in 4/25 (16%) patients at six months. At five years, only 2/17 patients (12%) reported anxiety and none had depression. From those who had anxiety at six months, two died, one was totally dependent for their daily activities, two recovered, and one continued to have high anxiety at the five-year follow-up. A new case with anxiety emerged in the extended follow-up. From those who had depression at six months, two recovered, one died, and one was completely dependent for their daily activities at the five-year follow-up. Psychiatric help after the first consultation was obtained for two patients with anxiety (one that was re-evaluated and another that was deceased at five years) and for one with depression (that died during the follow-up period).

At the six-month follow-up from ICU discharge, 4/25 (16%) patients presented signs of PTSD; two of these patients received psychiatric assistance, and one did recover. Five years later, none of the patients were identified with post-traumatic stress risk (two patients recovered, one died, and one was totally dependent for their daily routine activities and therefore not able to be evaluated) (Table 2).

The evaluation with EQ-5-D showed no significant differences in the following five domains: self-care, usual activities, pain/discomfort, mobility and anxiety/depression; additionally, no significant differences were found in the EQ VAS to quantify the state of health as follows: patients attributed a median (IQR) score of 60 (50 - 80) at six months *versus* 75 (50-80) at five years (p = 0.599).

## DISCUSSION

Cognitive impairment was found in 48% of the patients six months after ICU discharge, which is in accordance with previous studies (13 - 79% at the three- to six-month follow-up).^(14)^ Attention was the cognitive domain most frequently impaired in our cohort, followed by visual construction, memory and executive functions. Using other instruments, the literature points to attention, memory and executive functions as the most commonly affected domains in ICU survivors.^(4,7,14)^

Among the survivors, the recovery rate at five years was high (63%) in our study. This finding is in accordance with previous reports.^(14)^ In our study, visual construction was the only cognitive domain with a significant decline in the extended follow-up.

Regarding anxiety, depression and risk of PTSD, there was also a tendency towards resolution of symptoms over time, despite the patients' reluctance to obtain specialized help. Other studies point to a relative stabilization of the depressive symptoms in *acute respiratory distress syndrome* (ARDS) survivors over time.^(4,8)^ The risk of PTSD in our cohort at six month post ICU discharge was 16% in the lower range of that reported by other studies (12.5% to 63.6%); this variability could be related to different tools used to screen the disease, different cut-off values to define high risk of PTSD and a different mix of patients admitted into the ICU.^(15)^ Patients younger than 50 years, female gender and those with pre-existing psychological disease (PTSD or depression) present a higher risk for PTSD.^(15,16)^ Although we had a slight preponderance of male patients, 76% of our cohort was older than 50 years and we did not collect data on previous psychological disease, which could have contributed to the low prevalence reported.

Increasing age was associated with cognitive impairment (DRS-2 < 10^th^ percentile) six months after ICU discharge. Age is a recognized risk factor, and some of these patients may have already some kind of cognitive impairment that has not been previously diagnosed until the ICU admission that can prompt its recognition, deterioration or even be its cause.

Ideally, we should have a pre-ICU assessment of the patients' physical, cognitive and psychological status and quality of life to establish a baseline and then determine the true impact of the disease and/or ICU admission. These data are very difficult to obtain, because in the vast majority of cases, admission to the ICU is not anticipated. On the other hand, most of the tools used by clinicians to evaluate these domains are self-administered, cannot be applied to the next of kin (those more focused on cognitive and psychological status and quality of life) or are not validated for that purpose. Regarding physical activities, there are some instruments that can be administered to the next of kin, such as Lawton and Brody Instrumental Activities of Daily Living,^(17)^ and we proposed its application to the next of kin on patients' ICU admission.

A pre-hospital discharge evaluation would also be of great value to assess additional factors that could have arisen during hospital stays and contributed to the physical, psychological and cognitive impairments that remain at the follow-up clinic.

Patients with previous hypoxic episodes had less cognitive impairment. This finding is somewhat inconsistent with the literature. Severe hypoxic events are usually associated with cognitive impairment at one year follow-up, mainly in ARDS survivors, and less in patients admitted with general trauma.^(18)^ The counterintuitive results from our cohort could be explained by the lower age and lesser severity of acute illness of the patients with hypoxic events.

The use of sedative or analgesic medications was not significantly related with cognitive impairment. Pandharipande et al. also did not find a consistent association between the use of these medications and long-term cognitive impairment, but they found an association with delirium (at 12 months of follow-up).^(1)^ Unfortunately, daily screening for delirium was not fully implemented in our unit at the time of inclusion.

In the present study, glycemic dysregulation and severity of acute illness were not significant predictors of cognitive impairment. These negative findings are inconsistent with the literature. Sonneville et al. identified that persistent hyperglycemia was associated with significant neuronal and glial changes during critical illness and that preventing hyperglycemia was a neuroprotective strategy to protect against long-term cognitive impairment in survivors of critical illness.^(2)^ Hopkins et al. found that high blood glucose levels increased the odds of being cognitively impaired at one year.^(19)^ The severity of acute illness (based upon the Acute Physiology and Chronic Health Evaluation - APACHE - score) has been associated with cognitive impairment in small follow-up studies.^(14)^

To our knowledge, this is the first study to report results of a five-year follow-up after ICU discharge, in such a broad spectrum of domains, including cognitive and psychological functions and quality of life. Our results prompted the hypothesis that cognitive and psychological changes detected at 6 months after ICU discharge may recover in the very long term.

An important limitation of this study is the small number of patients included. As stated before, the lack of an evaluation before ICU admission precludes the conclusion that the alterations that were found are exclusively due to the acute illness and/or ICU admission, although the improvement observed in this cohort over time supports their potential impact in the impairment of the patients' cognitive and psychological functions and quality of life.

## CONCLUSION

Cognitive impairment is frequently observed after intensive care unit discharge. In our cohort, cognitive function, anxiety and depression improved significantly over time, suggesting the reversibility of these impairments over a significant period of time.

## References

[1] Pandharipande PP, Girard TD, Jackson JC, Morandi A, Thompson JL, Pun BT, Brummel NE, Hughes CG, Vasilevskis EE, Shintani AK, Moons KG, Geevarghese SK, Canonico A, Hopkins RO, Bernard GR, Dittus RS, Ely EW, BRAIN-ICU Study Investigators (2013). Long-term cognitive impairment after critical
illness. N Engl J Med.

[2] Sonneville R, Vanhorebeek I, den Hertog HM, Chrétien F, Annane D, Sharshar T (2015). Critical illness-induced dysglycemia and the
brain. Intensive Care Med.

[3] Hopkins RO, Jackson JC (2006). Long-term neurocognitive function after critical
illness. Chest.

[4] Adhikari NK, Tansey CM, McAndrews MP, Matté A, Pinto R, Cheung AM (2011). Self-reported depressive symptoms and memory complaints in
survivors five years after ARDS. Chest.

[5] Jones C, Griffiths RD, Slater T, Benjamin KS, Wilson S (2006). Significant cognitive dysfunction in non-delirious patients
identified during and persisting following critical illness. Intensive Care Med.

[6] Hopkins RO, Weaver LK, Collingridge D, Parkinson RB, Chan KJ, Orme Jr JF (2005). Two-year cognitive, emotional, and quality-of-life outcomes in
acute respiratory distress syndrome. Am J Respir Crit Care Med.

[7] Hopkins RO, Weaver LK, Chan KJ, Orme Jr JF (2004). Quality of life, emotional, and cognitive function following
acute respiratory distress syndrome. J Int Neuropsychol Soc.

[8] Hopkins RO, Key CW, Suchyta MR, Weaver LK, Orme Jr JF (2010). Risk factors for depression and anxiety in survivors of acute
respiratory distress syndrome. Gen Hosp Psychiatry.

[9] Boer KR, van Ruler O, van Emmerik AA, Sprangers MA, de Rooij SE, Vroom MB, de Borgie CA, Boermeester MA, Reitsma JB, Dutch Peritonitis Study Group (2008). Factors associated with posttraumatic stress symptoms in a
prospective cohort of patients after abdominal sepsis: a
nomogram. Intensive Care Med.

[10] Jurica PJ, Leitten CL, Mattis S, Cavaco Sara, Teixeira-Pinto Armando (2011). DRS-2 [Teste]: Escala de avaliação da demência - 2:
manual técnico = Dementia rating scale 2.

[11] Pais-Ribeiro J, Silva I, Ferreira T, Martins A, Meneses R, Baltar M (2007). Validation study of a Portuguese version of the Hospital Anxiety
and Depression Scale. Psychol Health Med.

[12] Twigg E, Humphris G, Jones C, Bramwell R, Griffiths RD (2008). Use of a screening questionnaire for post-traumatic stress
disorder (PTSD) on a sample of UK ICU patients. Acta Anaesthesiol Scand.

[13] Ferreira PL, Ferreira LN, Pereira LN (2013). Contribution for the validation of the Portuguese version of
EQ-5D. Acta Med Port.

[14] Wilcox ME, Brummel NE, Archer K, Ely EW, Jackson JC, Hopkins RO (2013). Cognitive dysfunction in ICU patients: risk factors, predictors,
and rehabilitation interventions. Crit Care Med.

[15] Griffiths J, Hull AM, Cuthbertson BH, Vincent JL (2008). Post-traumatic stress disorder in intensive care unit
survivors. Intensive Care Medicine: Annual Update 2008.

[16] Patel MB, Jackson JC, Morandi A, Girard TD, Hughes CG, Thompson JL (2016). Incidence and Risk Factors for Intensive Care Unit-related
Post-traumatic Stress Disorder in Veterans and Civilians. Am J Respir Crit Care Med.

[17] Lawton MP, Brody EM (1969). Assessment of older people: self-maintaining and instrumental
activities of daily living. Gerontologist.

[18] Guillamondegui OD, Richards JE, Ely EW, Jackson JC, Archer KR, Norris PR (2011). Does hypoxia affect intensive care unit delirium or long-term
cognitive impairment after multiple trauma without intracranial
hemorrhage?. J Trauma.

[19] Hopkins RO, Suchyta MR, Snow GL, Jephson A, Weaver LK, Orme JF (2010). Blood glucose dysregulation and cognitive outcome in ARDS
survivors. Brain Inj.

